# Research on the Influence of HMFI and PWHT Treatments on the Properties and Stress States of MAG-Welded S690QL Steel Joints

**DOI:** 10.3390/ma17143560

**Published:** 2024-07-18

**Authors:** Jacek Górka, Mateusz Przybyła

**Affiliations:** 1 Department of Welding Engineering, Silesian University of Technology, Konarskiego Str. 18a, 44-100 Gliwice, Poland; 2 FAMET S.A., Szkolna Str. 15, 47-225 Kędzierzyn Koźle, Poland; Mateusz.przybyla@famet.cpm.pl

**Keywords:** S690QL steel, MAG welding, technology qualification, peening, residual stresses

## Abstract

The aim of this study was to analyze the effect of the HFMI (high-frequency mechanical impact) treatment of each weld bead on the properties of a butt joint with a ceramic backing welded by robotic method 135 (MAG—metal active gas welding method) and to determine the effect of HMFI on the stress level. This analysis was based on a comparison of three butt joints made of a S690QL plate, in the as-welded condition, with the HFMI of each bead and with the heat treatment carried out with PWHT stress relief annealing. The high-frequency (90 Hz) peening of each weld bead was linked with a stress reduction in the weld via the implementation of compressive stresses into the joint. The HFMI pneumatic hammer was used for this. The correctness of treatment was achieved when 100% of the surface of each bead including the face was treated. As part of the post-welding tests, basic tests were carried out based on the standards for the qualification of welding technology, and as a supplementary test, a stress state analysis using the Barkhausen effect was carried out. The tests carried out showed that the use of high-frequency peening after each pass did not affect the negative results of all the required tests when qualifying the welding technology of S690QL sheet metal compared to the test plates in the as-welded condition and after heat treatment—stress relief annealing. Inter-pass peening of the welded face and HAZ (heat-affected zone) resulted in a reduction in post-weld residual stresses at a distance of 12 mm from the joint axis compared to the stress measurement result for the sample in the as-welded condition. This allowed for a positive assessment of peening in the context of reducing the notch, which is the concentration of tensile stresses in the area of the fusion line and HAZ. The tests carried out showed that the peening process does not reduce the strength properties of welded joints, and the results obtained allow the technology to be qualified based on applicable standards.

## 1. Introduction

The aim of this study was to analyze the effect of the HFMI treatment of each weld bead on the properties of a butt joint with a ceramic backing welded using a robotic method 135 (MAG) and to determine the effect of HMFI on the stress level and mechanical properties.

Welding techniques constitute the predominant discipline in global steel construction manufacturing. Concomitant with welding are the numerous technological challenges encountered by welding engineers on a daily basis [1]. Among these challenges, the main ones are the deformations and stresses that result from the welding processes; they are the phenomena induced by closely related and characteristic welding processes, encompassing phase transitions related to volume change, uneven and rapid heating and cooling, and alterations in properties such as Young’s modulus (E), yield strength (Re), or thermal expansion coefficient during heating and cooling [2,3,4,5]. The prevailing method employed to alleviate stresses and strains post-welding is heat treatment, notably stress relief annealing. This treatment utilizes electric or gas furnaces, or in localized applications, induction or resistance devices. Another method which can be implemented and is widely used is the optimization of welding parameters and sequences in order to reduce distortion [6]. An alternative to conventional post-weld heat treatments is hammer peening [7,8,9,10]. This method involves inter-pass peening or peening the face of welds, be they butt or fillet welds, to introduce compressive stresses through plastic deformations. The advantage of this method lies in its capability to facilitate both local and globally effective post-weld peening [11,12,13,14]. Peening can be executed through various systems and different conventional methods, such as electric or pneumatic impact weld dressing. Additionally, relatively recent methods of stress reduction by peening include “ultrasonic peening treatment” (UPT), “high-frequency impact treatment” (HiFIT), “ultrasonic peening” (UP), “pneumatic impact treatment” (PIT), and “ultrasonic needle peening” (UNP) [7,8]. These methods have primarily been developed to enhance impact efficiency, machining precision, and operator comfort by minimizing the impact on the operator [15,16,17,18,19]. European standards presently lack information regarding whether the use of high-frequency mechanical impact (HFMI) treatment is an essential variable in the welding process. Consequently, research is imperative to address key issues when qualifying metal arc welding technologies to meet the requirements of EN ISO 15614-1 and to examine the influence of HFMI on the results of the required tests [20]. Over the recent 70 years, the yield point of structural steel has surged by more than five times, commencing with low-alloy steel (Re about 200 MPa), progressing through higher-strength normalized low-alloy steel (Re about 350 MPa), steels produced with a thermo-mechanical treatment (Re up to 700 MPa), and culminating with quenched and tempered steels boasting a yield point of approximately 1300 MPa [21,22,23,24,25,26]. Fine-grained steels, as a classification, do not constitute a distinct group based on their production process, chemical composition, or mechanical properties. Rather, this designation pertains to steels characterized by a fine-grained microstructure in a condition set for delivery—a feature advantageous due to low grain growth in the heat-affected zone during the welding process [27,28,29,30]. Fine-grained steels are produced using normalizing, thermo-mechanical treatment, and quenching with tempering. The mechanical properties of fine-grained steels depend on both their chemical composition and the production process [31,32,33,34,35]. The primary challenge during the welding of quenched and tempered fine-grained steels is cold cracking. To optimize the strength and cracking resistance of a welded joint, it is imperative for the strength of the filler metal to be either equal to or slightly lower than the base material. The use of a filler metal with higher strength is not recommended. Welds should be strategically positioned in areas of construction with minimal stresses to mitigate the risks of cracking [36,37,38,39]. Currently, the hammering process has not been considered as one of the technological factors when qualifying welding technology. The tests carried out showed that the hammering process does not reduce the strength properties of the joints, and the results obtained allowed the technology to be qualified based on the applicable standards. Such knowledge is used in practice and allows in some cases to reduce the cost of heat treatment, especially in the case of repaired joints. The novelty of this research is based on a different approach compared to the well-known and already investigated fatigue strength improvement with high-frequency mechanical impact treatment. The direction of this research is based on the thesis that mechanical impact treatment can be considered as a partial replacement or complementary stress reduction method in terms of methods such as post-weld heat treatment, especially for materials where implementation of regular annealing has number of limitations such as S690QL. These limitations stem from the heat treatment state of delivery of quenched and tempered materials. Tempered steels in general can be subjected to stress annealing, adhering to the limits, which are in general 30–40 °C below the steel tempering temperature, which can cause a reduction in hardness and strength because it may temper the steel further, effectively softening it. If this limit is not adhered to, PWHT treatment carried out in a tempering range will result in a reduction in yield and tensile strength.

The aim of this article was to connect manufacturing approaches together with advanced and more detailed methodologies which can be implemented during the analysis of welding technology qualifications. Major research studies from a scientific point of view were focused on the determination of stress states while taking into the account that the method used (Barkhausen) could be implemented in heavy industry circumstances.

## 2. Materials and Methods

This analysis was based on a comparison of three butt joints made of S690QL plate (Table 1), in the as-welded condition, with the HFMI of each bead and with the heat treatment carried out via stress relief annealing. The consumable used for welding was Multimet brand solid IMT NiMoCr electrode wire with diameter Ø1.2 mm (Table 2).

### 2.1. Preparation of Welded Joints

**Figure 1:**
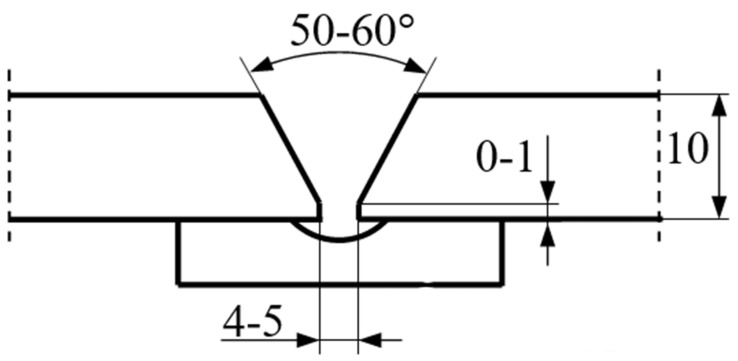
Weld joint preparation with ceramic backing.

**Figure 2:**
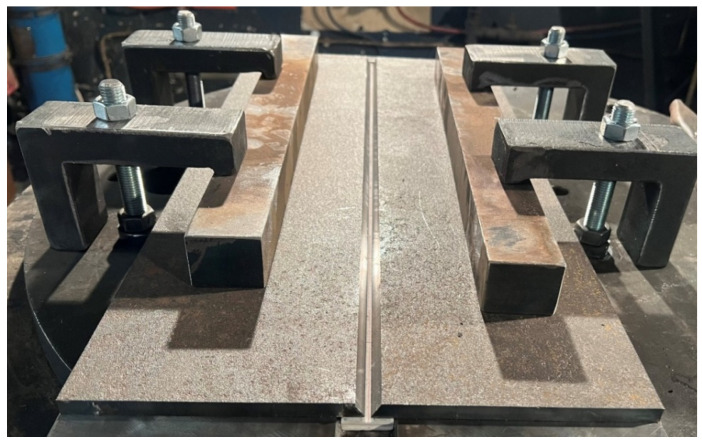
Test plate with ceramic backing on the welding robot bench.

As part of this work, 3 test plates were made of S690QL steel with dimensions of 10 × 150 × 600 mm. For each test plate, there were two plates with a 1/2 V beveled weld groove. These plates were mounted on a CLOOS robotic test bench together with a ceramic backing placed in the axis of the welded joint (Figure 1 and Figure 2).

The ceramic backing was utilized to ensure the accurate fusion and formation of the weld root on one side of the robotic bench. The welding procedure was executed on a CLOOS robotic workstation, guaranteeing uniform welding parameters across each of the test plates. These parameters encompassed arc voltage, welding current, number of stitches, welding speed, shielding gas composition (92% Ar + 8% CO_2_), and the stick-out distance from the welded element. The welding station operator meticulously recorded the welding parameters, and each plate underwent a three-layer welding process (refer to Table 3). The resultant outcomes of the average linear energy demonstrate that welding on the robotic workstation facilitated the attainment of consistent welding conditions concerning the linear energy of each bead.

### 2.2. High-Frequency Mechanical Impact

**Figure 3:**
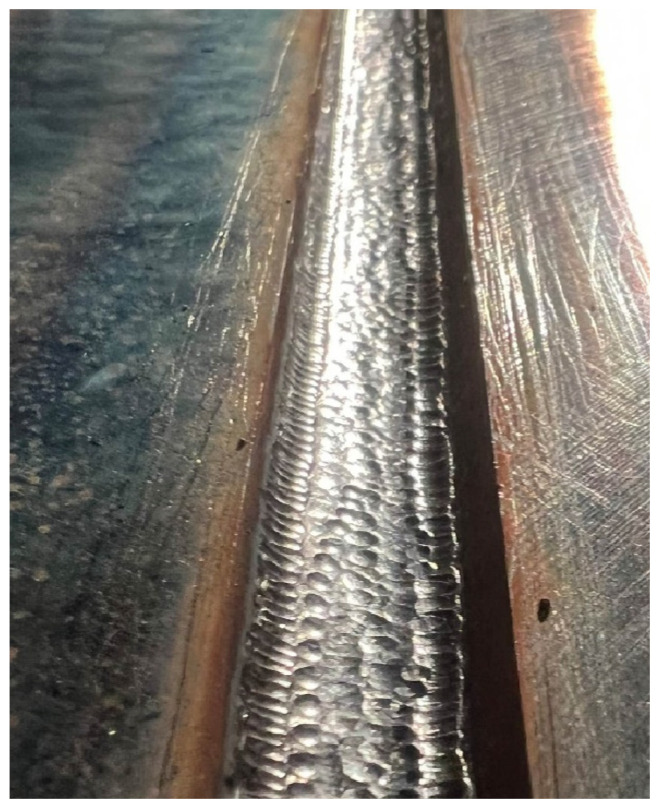
Welded bead surface after HMFI.

**Figure 4:**
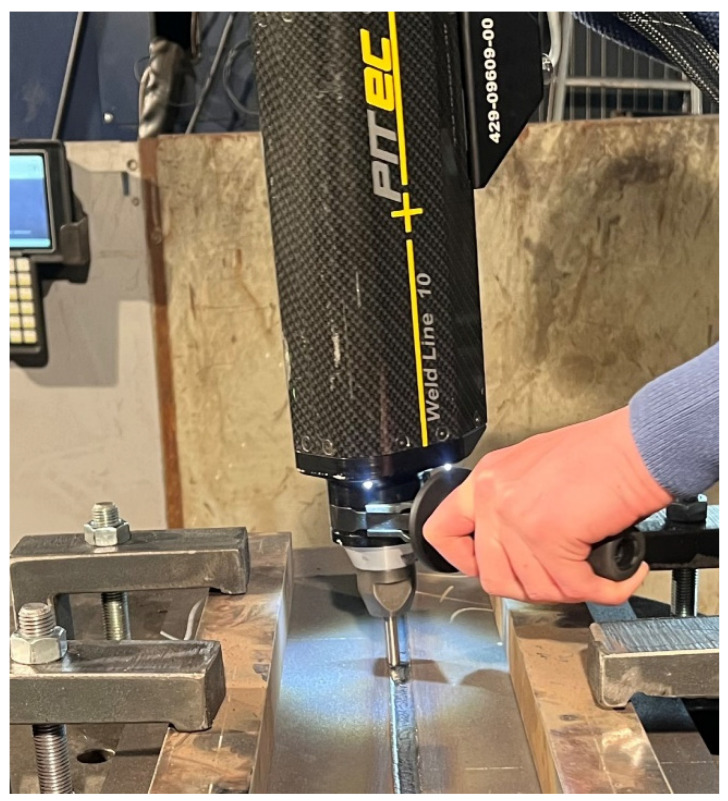
HFMI treatment of welding joint.

The high-frequency (90 Hz) peening of each weld bead was linked with stress reduction in the weld by implementation of compressive stresses into the joint. The Weld Line 10 pneumatic hammer from PITEC GmBH was used for this. The correctness of treatment was achieved when 100% of the surface of each bead including the face was treated (Figure 3). The HFMI was carried out manually (Figure 4).

### 2.3. Post-Weld Heat Treatment

**Figure 5:**
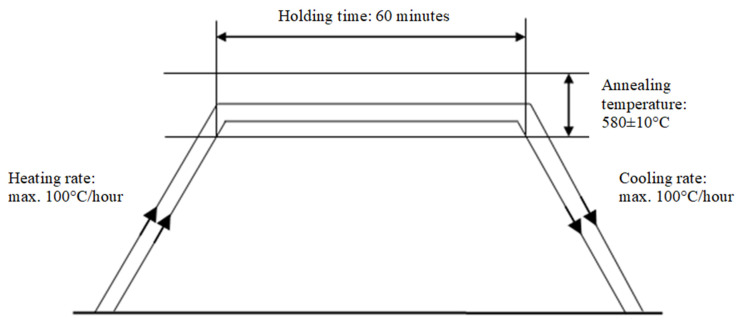
Post-weld heat treatment of S690QL test plate curve.

The third of the test plates after welding was subjected to heat treatment, which is commonly used to reduce the stress and deformation caused by welding processes. The stress relief annealing process in an electric furnace was divided into three stages: controlled heating, annealing, and controlled cooling (Figure 5).

### 2.4. Methodology of Tests and Acceptance Criteria

As part of the post-welding tests basic tests were carried out based on the standards for the qualification of welding technology, and as a supplementary test, a stress state analysis using the Barkhausen effect was carried out.

Tests were conducted in accordance with the requirements of EN ISO 15614-1:2017. In accordance with the requirements of the specification and qualification of metal welding technology (*Welding technology testing—Part 1*), each test plate was subjected to non-destructive testing:

⮚ Visual test (VT) in accordance with EN ISO 17637 [40];
⮚ Penetration test (PT) in accordance with EN ISO 3452-1 [41];
⮚ Radiographic testing (RT) in accordance with EN ISO 17636-1 [42].

All nondestructive tests on the test plates were positive.

The next step was to make specimens for destructive testing in accordance with EN ISO 15614-1:2017:

⮚ Tensile test—2 pieces of specimens according to EN ISO 4136 [43];
⮚ Side and root bend test—4 pieces of samples in accordance with EN ISO 5173 [44];
⮚ Charpy test—2 sets of samples in accordance with EN ISO 9016 [45];
⮚ Vickers hardness test—2 lines of measurement in accordance with EN ISO 9015-1 [46];
⮚ Macroscopic test—1 piece in accordance with EN ISO 17639 [47].

Due to the thickness of the base material, the impact test specimens were reduced to a dimension of 7.5 × 10 × 55 mm (so-called reduced cross-section specimens). For the transverse bending test, two pieces of 40 mm wide face bend specimens and two 40 mm wide rim bend specimens were made. Acceptance criteria for individual tests are shown in Table 4.

#### Barkhausen Effect Measurement

**Figure 6:**
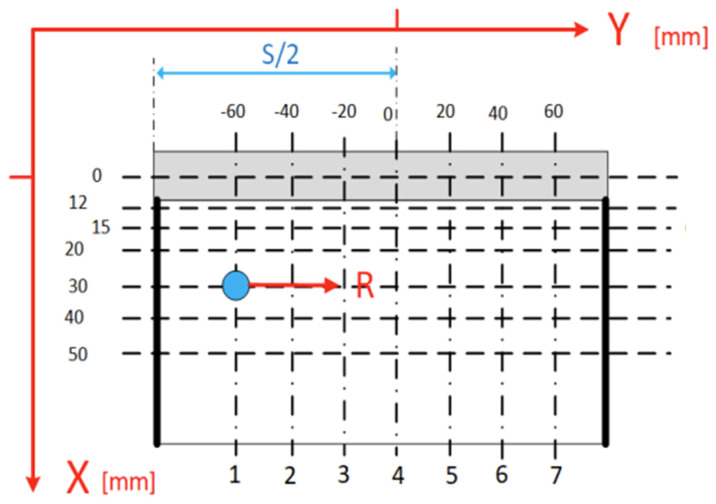
Location of measurement points in mm and test directions in relation to the welded joint.

The stress state was determined utilizing the MagStress5c (NNT Sp. z o.o. ul. Kartuska 432A, 80-125 Gdańsk, Poland) meter and a validated test procedure designed to ascertain this condition through the Barkhausen effect. This meter gauges the intensity of the Barkhausen effect by employing a standard probe (with a single core). The orientation of magnetization thereby dictates the orientation of the Barkhausen effect’s intensity examination. The recorded EB voltage signal is translated within the meter into an EB intensity descriptor, denoted as the INT parameter, serving as a quantification of the root mean square voltage of the EB voltage signal. Measurements of EB intensity, specifically the magnitude of INT, were conducted on the frontal aspect in the direction parallel to the weld axis (R direction) at various measurement points. These points were strategically positioned at the intersections of a grid formed by seven vertical and seven horizontal lines. The schematic representation of these points on the surface of the plate is illustrated in Figure 6. The initial horizontal line (designated as 0) aligned with the crest of the weld bead, while the vertical lines were sequentially numbered from 1 to 7, originating from the left edge of the weld. Stress measurements were calculated in three states: in the state after welding, after heat treatment (via stress relief annealing), and after peening.

The MagStress5c system autonomously translates the INT value into an epsilon (ε) strain through a calibration function table stored in its memory, represented as ε = FO(X). Here, X denotes the ratio of the measured INT intensity to the reference INT intensity value (INTref), assumed to signify the undeformed state (ε = 0). The meter logs both the INT value and the Si value, or stress (σ), calculated using the formula σ = E × ε, where E represents Young’s modulus. A constant value of E = 210 GPa is stored in the meter. In the current investigation, the adopted methodology involved the analytical computation of the ε strain value based on the recorded INT values within the gauge.

## 3. Results

The tests were performed in the destructive testing laboratory of FAMET Group S.A. in Kedzierzyn-Kozle. The laboratory was equipped with calibrated and verified equipment by accredited notified bodies in the field of destructive testing. Tests were completed with positive results for samples taken from the two welding test plates. Tensile test results were found negative for test plate samples taken from the PWHT specimen.

### 3.1. Destructive Tests Result Analysis

**Figure 7:**
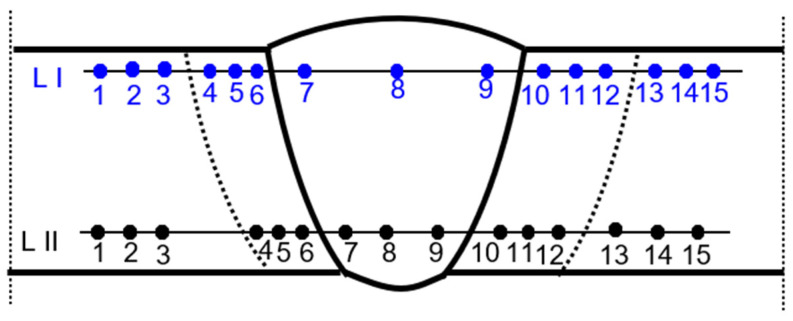
Location of the hardness test indentations in butt welds.

The results of destructive testing of welded joints are presented in Table 5, Table 6 and Table 7. The hardness measurement diagram is shown in Figure 7.

Transverse tensile test results for specimens taken from as-welded and HFMI-treated test plates met the requirements given and were within the acceptable limits. Results obtained from the post-weld heat-treated specimens taken from the test plate did not meet the requirements. The most probable reason for this is that the S690QL material was delivered in an as-tempered condition while exceeding 560 °C (PWHT was carried out at 580 °C—holding temperature). The result of initial tempering was altered so that the mechanical properties were negatively affected by the decrease in tensile and yield strength.

Face and root bend test results for all specimens were found within the acceptable limits, leading to the conclusion that welding, PWHT treatment, and HFMI treatment result in specimens having high plasticity, regardless of the technology used.

Impact tests results for all specimens were found to be within the acceptable limits. Results for the S690QL + PWHT heat-affected zones (average value 218.5 J) in comparison to the S690QL + HFMI (average value 68.1 J) zones displayed a higher value due to the lower values of hardness at both heat-affected zones, which were as follows: S690QL + PWHT (average value 206.6 HV10) and S690QL + HFMI (average value 270.6 HV10). Hardness test results for the S690QL + HFMI specimens showed that they displayed an increase in hardness in comparison with the post-weld heat-treated specimens: for HFMI, the average value was 301 HV10, while for PWHT, the average values was 270 HV10, signifying a 10.3% difference. Both the increase in hardness and the decrease in impact strength in the area subjected to HFMI were due to local intensive plastic deformations.

Macro specimens showed a compact and correct cross-section structure with the following zones being visible: parent materials, heat-affected zone, and weld. Macroscopic images revealed individual passes and boundaries between the primary segments of the welded joints. A fusion line was clearly visible, creating a boundary between the base material and heat-affected zone. For the specimen that was impacted mechanically at a high frequency, the treatment resulted in a concave transition between the weld face and the parent material. The concave transition in the weld toe area was subjected to additional measurements via a micro specimen visual examination after welding, and the HFMI treatment confirmed that the depth of the indentation was less than 0.5 mm, which qualifies this welding joint to at least of a quality level C (ISO 5817) [48]. The macro examination’s purpose was to detect potential inconsistencies (such as crack or pores); however, none were observed.

**Figure 8:**
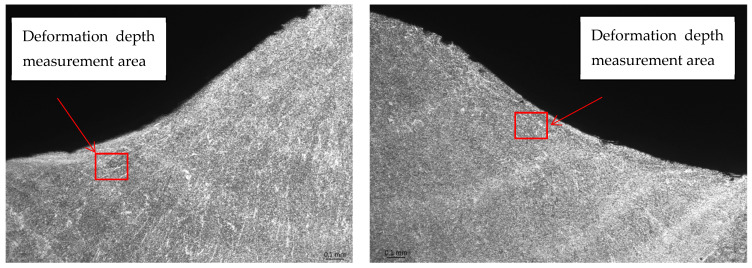
Microscopic examination of both sides of the weld in the HFMI area—magnification ×50.

**Figure 9:**
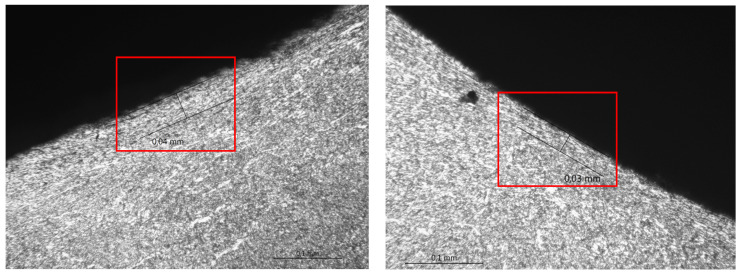
Microscopic examination of both sides of the weld in the HFMI area—magnification ×200.

In addition to a regular examination required by EN ISO 15614-1:2017, a micro examination was carried out in order to define what occurs after HMFI treatment (Figure 8 and Figure 9).

Figure 8 and Figure 9 show that due to the HFMI treatment being carried out during and after welding, the surface of the weld was impacted by a local deformation at a depth of 0.03–0.04 mm. No other consequences of a similar type could be observed in the area of the welded joint. According to ref. [7], a depth value of up to 0.3 mm was registered, but the authors were not provided any evidence for this, such as a macro/micro examination.

### 3.2. Stress Measurement Using the Barkhausen Effect

**Figure 10:**
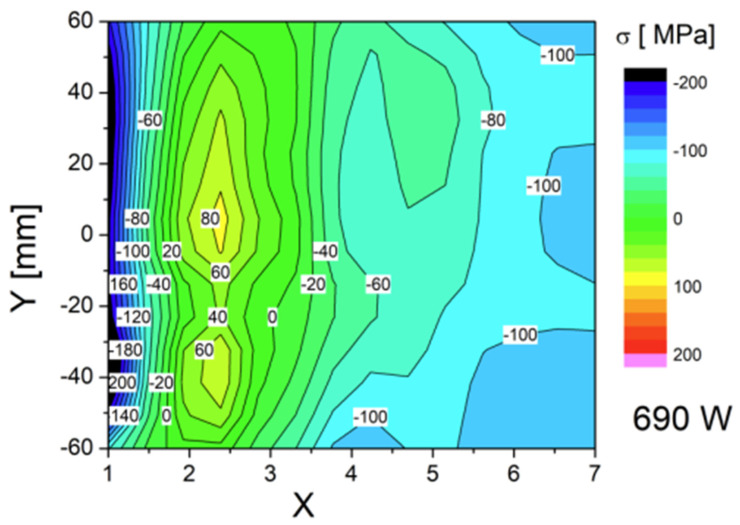
Stress distribution along the Y and X axes for the specimen after welding.

**Figure 11:**
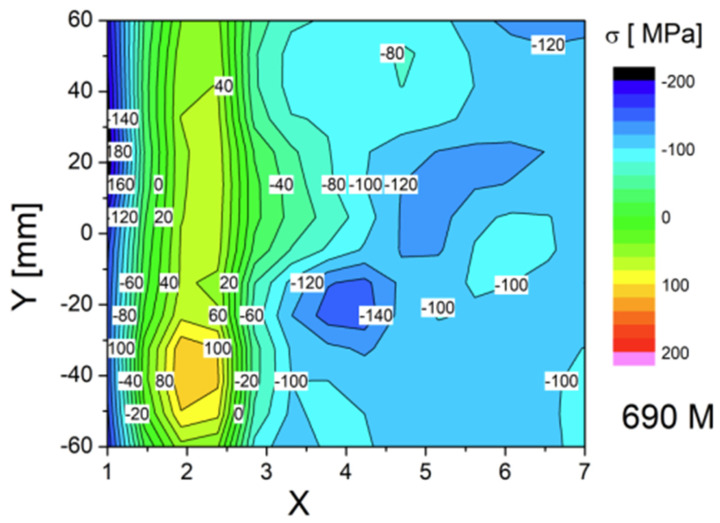
Stress distribution along the Y and X axes for the specimen after HFMI treatment.

**Figure 12:**
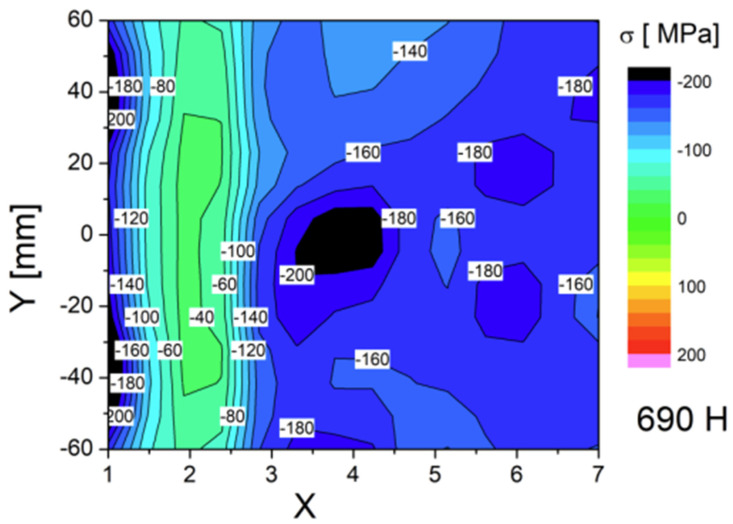
Stress distribution along the Y and X axes for the specimen treated with PWHT.

**Figure 13:**
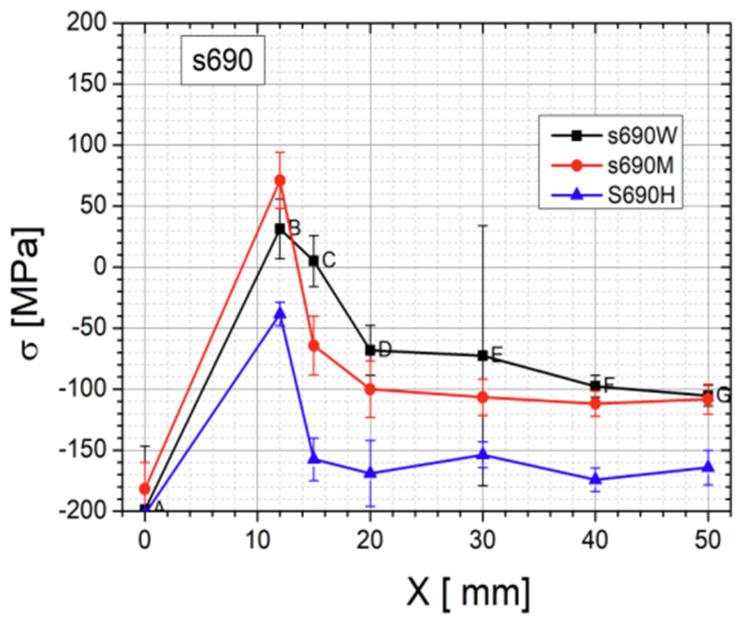
Summary comparing the measured stress values as a function of distance from the weld axis for all three test specimens: W—as-welded specimen; M—HFMI-treated specimen; and H, PWHT condition.

All three specimens were measured with the same number of measurement points (topography is shown on Figure 10), and the results were presented in the form of a color map—successive measurement points along the Y weld axis are depicted as a function of stress in MPa on the X axis (Figure 11, Figure 12 and Figure 13). For each side, three series of measurements were taken, and the distances from the weld face axis were, respectively, 0, 12, 15, 20, 30, 40, and 50 mm. Table 8 shows the summary of the results obtained—given as an INT value directly after the MagStress5c measurements were taken. After the conversion of the INT parameter, stress values were measured in MPa, and they are presented in Table 9.

From the graph shown in Figure 13, it can be seen that as a result of the welding process, the stress level in the region of the fusion line (12 mm from the weld axis) was about +30 to +70 MPa for the post-weld and HFMI specimens and displayed tensile stress. For the PWHT-treated sample, its average value according to the the measurements was about −40 MPa, and it displayed compressive stress. At the measurement points located in and around the HAZ (15 mm from the weld axis), the stress for the post-weld sample was +5 MPa, while it was −65 MPa for the post-HFMI sample and −140 MPa for the PWHT sample. The results in these areas illustrate what changes occur as a result of welding processes and subsequent treatments, such as HFMI or the PWHT. To comprehensively characterize the impacts of the annealing procedure on the stress distribution concerning the distance from the weld axis (X), mean values derived from sigma (σ) values and corresponding standard deviations (∆σ) were computed for seven specific points situated along a designated line. These calculated values are presented in Table 10 below.

The type and state of residual stress in each of the controlled specimens allows us to summarize from the weld’s integrity and structural performance that there was a significant decrease observed in terms of properties such as tensile strength in the post-weld heat-treated welding joint. Table 10 confirms that a significant change in the joint’s stress state occurred. Based on the mechanical tests and Barkhausen stress measurements, it can be observed that for the as-welded joint, the stress level was higher in comparison to as-HFMI-treated one; nonetheless, both welding joints passed the mechanical tests. Based on the structural performance of the joints and on the other research studies analyzed, HFMI had a positive impact on cyclic loading and fatigue conditions.

## 4. Summary

The conducted tests lead to the following conclusions:

⮚ Based on this article, it was proven that a classic welding technology qualification can be carried out together with HFMI as it has no negative impact on the obtained results.
⮚ Tensile test results were positive for specimens taken from as-welded and HFMI-treated weld samples. For the PWHT-treated sample, the tensile test showed a value that decreased below the requirement. It showed that the PWHT of quenched and tempered construction steels has a negative impact on tensile strength. On the other hand, the results showed that HMFI did not reduce their mechanical properties.
⮚ Face and root bending tests showed no inconsistencies and met the acceptance criteria specified in the standards.
⮚ Charpy test results were within the range of acceptance criteria for all three samples. The HFMI sample displayed the lowest values in both the HAZ and weld area compared to as-welded and PWHT-treated samples.
⮚ Hardness measurements showed that using HMFI caused a hardness increase in the area that was treated. The reason for this is that a local high plastic deformation occurs due to hammer peening. Despite the increase in hardness in the HFMI sample, the values were still within the outlined limits.
⮚ The macroscopic examination showed no inconsistencies on the cross-sections of all three test plates.
⮚ The spatial distribution of the stress level specific to each treatment condition was revealed. The stress state in the HAZ zone was particularly interesting. The authors believe that the HAZ zone can be considered as being 12–15 mm away from weld axis (Figure 13).
⮚ The Barkhausen measurements showed that the HFMI treatment has a positive impact on the HAZ in comparison to as-welded sample but that the benefit is lower compared to regular PWHT. The PWHT conducted was slightly above the tempering temperature, and this is a reason why HFMI was less beneficial in comparison to annealing in terms of a reduction in residual stress.
⮚ Evidently, the high-frequency mechanical impact (HFMI) treatment markedly diminishes stress levels within welds. An analysis of the stress distribution plots presented in Figure 13, depicting as-welded and HFMI-treated states with respect to the distance on the X axis, further allows us to infer that for distances of X ≥ 15 mm, the impact of HFMI is virtually negligible.
⮚ The results indicate that a further investigation in terms of HFMI treatment with regard to a reduction in stress states is necessary and will be carried out by the authors in the future.

## Figures and Tables

**Table 1:** Chemical composition of welded base material (plate) from Material Certificate 3.1.

Plate	Element Concentration, wt %													
	C	Mn	Si	P	S	Cr	Mo	Ni	V	Cu	Al	Ti	Nb	Zr
S690QL	0.143	1.262	0.285	0.01	0.0006	0.259	0.302	0.823	0.004	0.087	0.028	0.001	0.023	0.001

**Table 2:** Chemical composition of IMT NiMoCr wire from Material Certificate 3.1.

Wire	Element Concentration, wt %											
	C	Mn	Si	P	S	Cr	Ni	V	Cu	Al	Mo	Ti + Zr
IMT	0.082	1.60	0.56	0.01	0.006	0.347	1.43	0.09	0.02	0.002	0.27	0.002

**Table 3:** Welding parameters of test plates (L = 600 mm).

Sample Designation	Bead No	Inter-Pass Temperature [°C]	Average Welding Current [A]	Average Arc Voltage [V]	Welding Time [min]	Linear Energy [kJ/mm]
S690QL As welded	1	28.3	194	24.3	2:46	1.05
	2	82.5	200	27.8	1:36	0.71
	3	104.5	247	26.5	2:31	1.32
S690QL + HMFI	1	34.0	192	24.2	2:44	1.02
	2	60.6	202	27.4	1:37	0.72
	3	108.5	250	26.4	2:29	1.31
S690QL + PWHT	1	36.0	195	24.3	2:44	1.04
	2	92.4	199	27.3	1:38	0.71
	3	119.7	249	26.5	2:33	1.35

**Table 4:** Acceptance criteria for all tests.

Test Type	Acceptance Criteria [12]
Tensile test	R_m_ value should not be less than the corresponding required minimum value for the base material—R_m_ minimum 770 MPa
Bend test	During the test, there should be no inconsistencies in the specimens above 3 mm in any direction—bending former radius 60 mm
Impact test	Impact value shall be in accordance with the relevant standard of the base material—KV_2_ minimum 40 J at −20 °C
Hardness test	For non-heat-treated specimens—HV10 max. 450 For heat-treated (PWHT) specimens—HV10 max. 380
Macroscopic examination	No nonconformities in quality levels lower than those described in Table 4 of EN ISO 15614-1:2017-08 [20]

**Table 5:** Test results for as-welded joints.

Type of Test	Designation	Result					Designation						Result				
Tensile test	TT-1	793 MPa					TT-2						791 MPa				
Face bend test	TFBB1	positive					TFBB2						positive				
Root bend test	TRBB1	positive					TRBB2						positive				
Impact test	VWT 0/2	122.6 J					VHT 0/2						73.6 J				
	VWT 0/2	130.8 J					VHT 0/2						85.0 J				
	VWT 0/2	135.7 J					VHT 0/2						62.1 J				
Hardness test	Ma-1 HV10	L1	263	268	264	195	197	234	272	271	268	207	188	234	250	261	266
		L2	264	261	258	201	193	225	266	265	256	216	186	199	251	261	258
Macroscopic examination	Ma-1	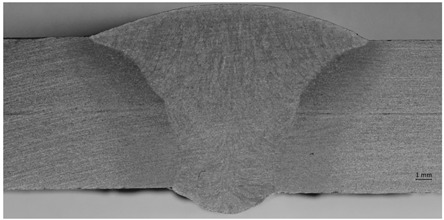															

**Table 6:** Test results for HMFI-treated welding joints.

Type of Test	Designation	Result					Designation						Result				
Tensile test	TT-1	782 MPa					TT-2						780 MPa				
Face bend test	TFBB1	positive					TFBB2						positive				
Root bend test	TRBB1	positive					TRBB2						positive				
Impact test	VWT 0/2	85.0 J					VHT 0/2						71.9 J				
	VWT 0/2	114.5 J					VHT 0/2						65.4 J				
	VWT 0/2	94.8 J					VHT 0/2						67.0 J				
Hardness test	Ma-1 HV10	L1	275	267	267	268	248	311	293	298	313	310	300	267	267	271	278
		L2	284	285	263	236	262	250	298	287	296	272	261	262	274	278	277
Macroscopic examination	Ma-1	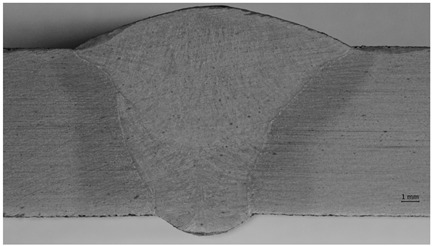															

**Table 7:** Test results for post-weld heat-treated welding joints.

Type of Test	Designation	Result					Designation						Result				
Tensile test	TT-1	714 MPa (negative)					TT-2						714 MPa (negative)				
Face bend test	TFBB1	positive					TFBB2						positive				
Root bend test	TRBB1	positive					TRBB2						positive				
Impact test	VWT 0/2	101.4 J					VHT 0/2						225.6 J				
	VWT 0/2	114.5 J					VHT 0/2						228.9 J				
	VWT 0/2	85.0 J					VHT 0/2						201.1 J				
Hardness test	Ma-1 HV10	L1	264	268	264	195	197	234	272	271	268	207	188	238	250	261	266
		L2	264	261	258	201	193	225	266	265	256	216	186	199	251	261	258
Macroscopic examination	Ma-1	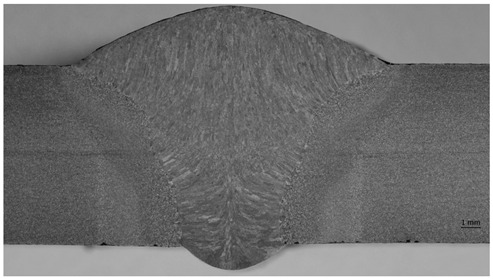															

**Table 8:** INT parameter values measured on all three test samples.

As-Welded S690QL	0 mm	12 mm	15 mm	20 mm	30 mm	40 mm	50 mm
1	1967	3906	2456	1769	1861	1784	1810
2	1268	4852	3340	2033	1946	1774	1799
3	1388	4010	3549	2327	2008	1910	1859
4	1406	4988	4335	2095	2112	1896	1750
5	1362	4741	3951	2089	2229	1896	1810
6	1350	4207	3987	2167	2293	1926	1902
7	1464	3094	3602	2356	2007	1829	1742
**HFMI-Treated S690QL**	**0 mm**	**12 mm**	**15 mm**	**20 mm**	**30 mm**	**40 mm**	**50 mm**
1	1497	4876	1840	1864	1774	1742	1906
2	1561	5674	2057	1796	1778	1756	1895
3	1568	5136	1939	1575	1823	1831	1731
4	1511	5031	2601	1956	1692	1878	1773
5	1372	5003	2607	1918	1714	1707	1774
6	1402	5010	2083	1948	1960	1795	1796
7	1447	4663	2172	1939	1912	1704	1697
**S690QL + PWHT**	**0 mm**	**12 mm**	**15 mm**	**20 mm**	**30 mm**	**40 mm**	**50 mm**
1	1517	2276	1510	1457	1632	1536	1560
2	1276	2594	1552	1558	1544	1507	1499
3	1396	2619	1478	1480	1550	1452	1639
4	1457	2602	1503	1369	1563	1498	1499
5	1466	2619	1667	1536	1522	1458	1548
6	1318	2465	1605	1636	1578	1519	1464
7	1516	2240	1638	1650	1646	1539	1556

**Table 9:** Stress state values after conversion from INT into MPa on all three test samples.

As-Welded S690QL	0 mm	12 mm	15 mm	20 mm	30 mm	40 mm	50 mm
1	−82	16	−39	−112	−97	−109	−105
2	−261	56	−3	−74	−85	−111	−107
3	−211	19	4	−48	−77	−90	−97
4	−205	64	31	−68	−66	−92	−115
5	−221	50	17	−68	−55	−92	−105
6	−226	26	19	−61	−50	−88	−91
7	−185	−11	6	−46	−77	−102	−117
**HFMI-Treated S690QL**	**0 mm**	**12 mm**	**15 mm**	**20 mm**	**30 mm**	**40 mm**	**50 mm**
1	−175	57	−100	−96	−111	−117	−90
2	−157	124	−72	−107	−110	−114	−92
3	−155	74	−86	−154	−103	−102	−119
4	−171	67	−31	−84	−127	−94	−111
5	−217	65	−31	−89	−122	−124	−111
6	−206	65	−69	−85	−83	−107	−107
7	−191	46	−60	−86	−89	−124	−126
**S690QL + PWHT**	**0 mm**	**12 mm**	**15 mm**	**20 mm**	**30 mm**	**40 mm**	**50 mm**
1	−169	−52	−171	−188	−140	−164	−158
2	−257	−32	−160	−158	−162	−172	−175
3	−208	−30	−181	−180	−160	−189	−138
4	−188	−31	−173	−218	−157	−175	−175
5	−185	−30	−132	−164	−168	−187	−161
6	−239	−39	−146	−139	−153	−169	−185
7	−170	−55	−138	−136	−137	−163	−159

**Table 10:** Stress state values after conversion.

X [mm]	As-Welded S690QL	Δσ	690QL + HFMI	Δσ	690QL + PWHT	Δσ
0	−199	52	−182	22	−202	32
12	31	24	71	23	−38	10
15	5	21	−64	24	−157	17
20	−68	20	−100	23	−169	27
30	−73	−107	−107	15	−154	11
40	−98	9	−112	10	−174	10
50	−105	9	−108	12	−164	14

## Data Availability

The data presented in this study are available upon request from the corresponding author. The data are not publicly available because the authors do not wish to publish supplementary materials.
